# Assessment of community-based intervention approaches to improve the health and welfare of working donkeys in selected areas of Sidama region, Southern Ethiopia

**DOI:** 10.3389/fvets.2023.1253448

**Published:** 2024-01-22

**Authors:** Aweke Yalew, Daniel Darge, Berhanu Mekibib Melake

**Affiliations:** ^1^Wolaita Sodo Agricultural Technical Vocational and Educational Training College, Wolaita Sodo, Ethiopia; ^2^Department of Veterinary Medicine, Faculty of Veterinary Medicine, Hawassa University, Hawassa, Ethiopia

**Keywords:** animal based assessment, working donkeys, healthcare, community-based intervention, welfare

## Abstract

**Background:**

Although efforts have been made by certain non-governmental organizations, like the Donkey Sanctuary, SPANA (Society for the Protection of Animals Abroad), and Brooke Ethiopia, to change the attitudes and practices of donkey owners toward improving the health and welfare of working donkeys, their impact has not been assessed so far. A cross-sectional study was conducted to assess and compare donkeys’ health and welfare problems in community-based intervention areas versus non-intervention areas in selected districts of Sidama regional state, Southern Ethiopia.

**Methods:**

For the animal-based welfare assessments, 200 donkeys each were selected and included from intervention and non-intervention areas. The selected donkeys were then assessed for their welfare and health status using five important parameters, namely, body condition score, presence and severity of wounds, behavior, presence and severity of lameness, and presence of other signs of illness/diseases.

**Results:**

The prevalence and severity of lameness and wounds on donkeys managed in non-intervention areas were higher than those observed in community-based intervention areas. The prevalence of lameness in the non-intervention areas (25.5%) was over two times higher than the prevalence in the intervention areas (12%). Likewise, over 37% of the donkeys in the non-intervention areas were wounded, of which 64% were suffering moderate to severe wounds. Moreover, donkeys in the intervention areas had better body condition and were alert and friendly upon human approach. There was a statistically significant difference (*p* < 0.01) between the intervention and non-intervention areas in all the considered parameters, namely, the presence of lameness, wound, body condition score, demeanor, and response to approach.

**Conclusion and recommendations:**

Based on this study’s findings, the community-based intervention approach was found to improve the health and welfare of working donkeys. Therefore, comprehensive and continuous equine health and welfare promotion through community-based intervention approaches should be designed and implemented to improve the welfare of working equines in the country.

## 1 Introduction

Ethiopia has approximately 10.80 million donkeys, the largest population in Africa and the second largest in the world. Nearly 47% of the country’s total donkey population is male and nearly 0.61% (65,282) are found in Sidama regional state (1). These donkeys, particularly the male ones, play a significant role as draft power and transport for goods in rural, peri-urban, and urban areas throughout the year (2, 3). According to the report of Admasu and Shiferaw (4), approximately 56% of donkeys are kept mainly for pack services (to generate income and for homestead use), 26% for cart use (to generate income), 14% for pack use but exclusively for homestead use, and 4% exclusively for renting, breeding, or petty trade.

Poor infrastructure and very rugged topography in many parts of rural Ethiopia and many other developing countries have made transportation by vehicle inaccessible. Hence, people in rural and peri-urban areas rely on equines to transport crops, fuel wood, water, and people by cart or on their backs from farms and/or markets to homes. The donkey is one of the most important equine draught animals, playing a key role in the agricultural economy (5). As the beast of burden, donkeys are good vehicles and will remain the main means of transport in the mountainous and rugged landscapes of Low-Middle income countries, where motor road construction is difficult (6) and where households are poorly resourced (7, 8).

Compared to horses, donkeys are assumed to be hardy; hence, they often engage in work for long hours without proper management, which in turn negatively affects their health, welfare, and quality of life. Moreover, donkeys in Ethiopia are the most neglected animals due to low social status (9). This was partly justified by the low number of sick donkeys presented annually to the veterinary clinics compared to other domestic animals (10). The prevailing misuse and mistreatment of donkeys and the lack of veterinary care for donkeys have contributed enormously to early death and work life expectancy of 4–6 years. However, in countries where animal welfare is in practice, the life expectancy of donkeys reaches up to 30 years (11).

Owing to the prolonged and strenuous work they carry out and the little attention they receive (low social status), donkeys in developing countries are subjected to a variety of health and welfare problems, including back sores and other wounds, lameness, colic, parasitosis, and various infectious diseases such as tetanus, strangle, epizootic lymphangitis, etc. (12–15). Apart from these, there are several other interacting factors affecting the welfare outcomes of working equids (16), the majority of which can be improved through community awareness creation.

Since the beginning of 1994, three foreign non-governmental organizations, namely, The Donkey Sanctuary, SPANA, and Brooke Ethiopia, have been intensively working in the country to improve the health and welfare of equines by launching outreach treatment centers and community-based intervention approaches. The outreach treatment approach was to directly provide the health and veterinary services and provide some material support, whereas the community-based approach was to develop the capacity of the community to improve the welfare of their donkeys by keeping their donkeys healthy, preventing wounding and lameness through education and training, supporting veterinary services, and training farriers and pack saddle and harness makers. Community-based interventions also include promoting practices such as wound management, eye cleaning, watering, feeding, grooming, and hoof picking and discouraging practices like beating and overloading through the training of change agents (elementary school children, teachers, and voluntary community champions for equine welfare).

Brooke Ethiopia and The Donkey Sanctuary focused primarily on working donkeys through the provision of treatment for sick donkeys and different harness materials, better carts, and implements for resource-poor owners, as an outreach treatment approach. However, in the latter stage, both non-governmental organizations adopted community-based approaches/interventions whereby change agents (model equine owner/users) and school children receive tailored training and education to work jointly with these non-governmental organizations as an advocacy group and/or concomitantly transfer their skills and knowledge to peers, which then widely trickles down to other equine owners or users of the area (17, 18).

Although very few studies have been conducted to assess the welfare of working equines (8, 19–23) and to compare the effectiveness of different knowledge-transfer interventions for rural working equid users (24), information is lacking on the impact of the interventions made so far on the community regarding the improvement of the health and welfare of working donkeys in Ethiopia. Therefore, the study aims to assess the impact of community-based interventions on improving donkeys’ health and welfare status.

## 2 Materials and methods

### 2.1 Description of study area

**Figure 1:**
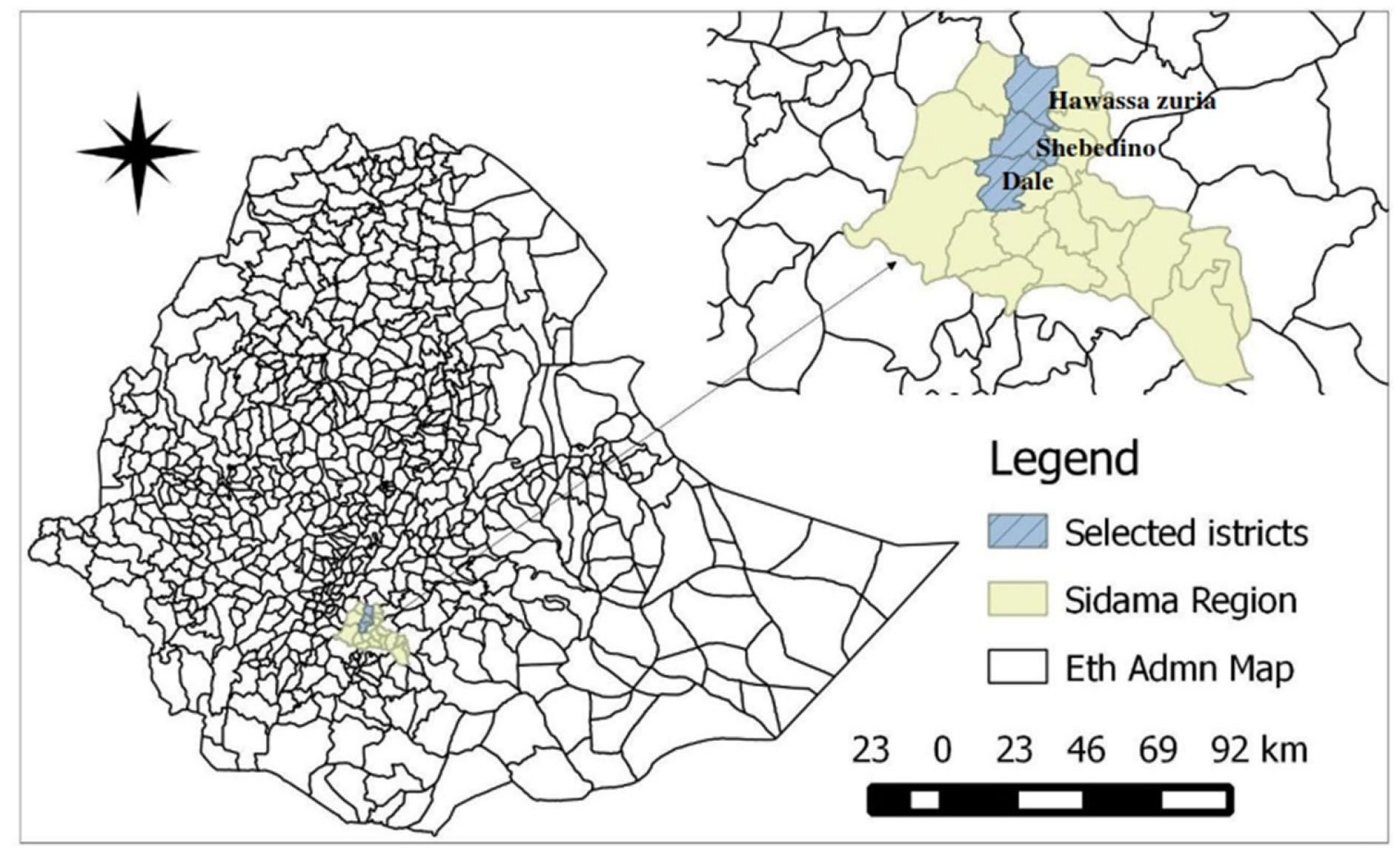
Map of the selected districts (developed using QGIS software version 2.01).

The study was conducted in three districts of Sidama regional state, namely, Hawassa (Hawassa Zuria district), Leku (Shebedino district), and Yirgalem (Dale district), Southern Ethiopia (Figure 1). These areas were selected because they are among the community-based intervention areas of The Donkey Sanctuary Ethiopia; a United Kingdom-based international non-governmental organization. The three study areas, namely, Hawassa, Leku, and Yirgalem are located 270, 295, and 310 km south of Addis Ababa, respectively, at an altitude range of 1,790–2,950 m.a.s.l. The selected districts are known for their high donkey population and highly dense populations and are commonly considered representative of the region’s socio-economic and cultural issues (25). The climate in the zone is characterized by a long rainy season from June to September, accounting for 75% of total rainfall; a short rainy season ranging from February to May; and a dry season from October to January (26).

### 2.2 Study population and animals

The donkey populations in the study area are dominated by male indigenous donkeys purchased from different markets in the region. The donkey population in the three districts (woredas) is estimated to be 13,442 in Dale, 13,751 in Shebedino, and 18,400 in Hawassa Zuria district (Hawassa, 2015, unpublished). As the only affordable and feasible transport system for the undeveloped road infrastructure in the area, donkeys transport various goods, such as construction materials (building blocks, sand, gravel, and stone), agricultural products and by-products (from the field to homes and then to market), firewood, and drinking water. Cart-pulling donkeys occupy the main livelihood and income-generating activities for the majority of the owners of the animals. The feeding system for donkeys involves supplemental feeding with wheat bran, chopped sugar cane tops, and household food waste during working time and grazing on communal lands or foraging through trash on roadsides (18, 27).

All the donkeys included in the study were male, working, and were managed along with other domestic animals. The majority of the donkeys in the intervention and non-intervention areas are used for cart pulling and are within the age range of 6–15 years. The selected donkeys were managed (fed, watered, housed, cleaned, and used) by the owners (78%) or by daily laborers hired to work for the owners (22%). The majority of these individuals were between 15 and 30 years old, at elementary school level, married and has 4 or more family members who are dependent on their donkey (Table 1).

**Table 1:** Demographic details of the study animals and their owners/users.

Variable		Intervention areas, no (%)	Non-intervention areas, no (%)	Total no (%)
Work type	Cart	189 (94.5)	101 (50.5)	290 (72.5)
	Pack	11 (5.5)	96 (48)	107 (26.75)
	Both	0	3 (1.5)	3 (0.75)
Age of Donkey	3–6 yrs	22 (11)	22 (11)	44 (11)
	6–10 yrs	81 (40.5)	88 (44)	169 (42.25)
	10–15 yrs	71 (35.5)	76 (38)	147 (36.75)
	>15 yrs	26 (13)	14 (7)	40 (10)
Donkey ownership	Owner	154 (77)	158 (79)	312 (78)
	Hired/commission	46 (23)	42 (21)	88 (22)
Educational status	Illiterate	70 (35)	65 (32.5)	135 (33.75)
	Elementary school	112 (56)	112 (56)	224 (56)
	High school	18 (9)	23 (11.5)	41 (10.25)
Marital status	Single	27 (13.5)	27 (13.5)	54 (13.5)
	Married	166 (83)	162 (81)	328 (82)
	Divorced	7 (3.5)	11 (5.5)	18 (4.5)
Age of the owner/user	10–15 yrs	37 (18.5)	42 (21)	79 (19.75)
	15–30 yrs	101 (50.5)	122 (61)	123 (55.75)
	30–60 yrs	62 (31)	36 (18)	98 (24.5)
Number of dependent family members	1–3	22 (11)	24 (12)	46 (11.5)
	4–6	120 (60)	116 (58)	236 (59)
	7–10	58 (29)	60 (30)	118 (29.5)

### 2.3 Sample size determination

The total sample size required for this study was determined using Slovin’s (Yamane’s) formula, as follows:


$$n=\frac{N}{1+N{\left(0.05\right)}^{2}}$$

Where n is the sample size, N is the population size of donkeys in the three study districts, and 0.05 is the level of precision or the sampling error. Using the total number of 45,593 donkeys in the three districts, the total number of donkeys for the study was calculated to be 396.52, which was then rounded up to 400.

### 2.4 Study design and sampling strategy

A cross-sectional study design was employed, involving 400 pack and cart donkeys selected from three selected districts (Hawassa, Dale, and Shebedino) of Sidama regional state. During sampling, the kebeles (smaller administrative units) in each town, which were covered by the intervention, were selected and included in the study. Non-governmental organization intervention began approximately 10 years ago in the selected kebeles (Shell, Remeda, and Bera). Similarly, an equal number of kebeles from non-intervention areas that are far from and do not share common market/other communal places with donkey owners from the intervention areas (namely Fichawa, Amfarara, and Megara) were systematically drawn as the cohort. Then, working donkeys in the selected kebeles were systematically selected using a non-probability sampling technique and included in the study regardless of work type, body condition, sex, and age (28). The selection was made on a door to door basis. When a household was found with more than one donkey, only one was selected for the assessment. Accordingly, 200 donkeys were selected from the intervention areas and 200 from non-intervention areas. The consent of animal owners was requested before each sampling procedure. If the donkey owner was not willing, the opportunity was given to the next willing donkey owner.

### 2.5 Study methodology

A structured direct assessment format was developed, and data was collected from November 2019 to July 2020 by direct physical examination of the donkeys. During off-hours (6:00–8:00 AM and 5:00–7:00 PM), the donkeys were thoroughly examined by the first author (a veterinarian who received short training on animal welfare) for the general health condition and welfare status. The parameters used for assessing the welfare and health of the selected donkeys were body condition score, presence of wounds and lameness, behavior, and other signs of illness/diseases. Body condition was assessed and scored using a body condition scoring system developed and used by The Donkey Sanctuary (1–5, 1 = poor/emaciated and 5 = obese/very fat). For ease of analysis and presentation, the body condition scores (BCS) were recently named as poor (BCS less than or equal to 2), medium (BCS 3), and good (BCS equal to or above 4).

Any active wound on any part of the external body was characterized and recorded considering the respective anatomical location (29). Based on the recommendation of Sells et al. (30), the wounds encountered were numbered and scored objectively according to their severity (Table 2). Accordingly, an ‘overall wound score (OWS)’ was calculated for each donkey by summing the products of the number of wounds and the grades in each wound category (e.g., 3 × Grade 1 wounds [3] + 1 × Grade 2 wound [2] = 5).

**Table 2:** Scoring system for wound severity.

	Wound score	Description
Superficial	1	Hair has been rubbed off; skin is not broken, but the lesion is painful when palpated or the skin is broken
Medium	2	Subcutaneous tissues are visible and damaged.
Deep	3	Muscle layers or bone visible.

Source: Sells et al. (30).

Lameness (gait abnormality) was assessed by watching the donkey walk forward for approximately 12 steps, with the researchers observing from behind and the side (15). The encountered lameness was characterized and scored on a scale of 0 (sound) to 5 (non-weight bearing), as indicated by the American Association of Equine Practitioners (31) (Table 3). Moreover, each limb, joint, and foot of these donkeys were examined for any lesion noted and palpated at rest.

**Table 3:** Grades of lameness with their respective descriptions.

Grade 0	Sound/no detectable lameness under any circumstances.
Grade 1	Lameness that is difficult to observe and is inconsistently apparent regardless of the circumstances (e.g., in hand or under saddle, hard surface, incline).
Grade 2	Difficult to detect at walk or trot in a straight line, but is consistently apparent under particular circumstances (e.g., under saddle, hard surface, incline).
Grade 3	Lameness is consistently observed at a trot in all circumstances.
Grade 4	Lameness is obvious with a marked head nod, hip hike, and/or shortened stride.
Grade 5	Lameness is obvious with minimal weight bearing either during motion or at rest. The animal might be unable to move.

Source: AAEP (31).

A behavioral assessment was conducted to measure general attitude (alert or dull), and response to observer approach and handling (difficult to catch or friendly) according to Hausberger et al. (32). Each donkey was thoroughly examined following the general physical examination schemes (inspection, palpation auscultation, and percussion) for the presence of any clinical signs suggestive of disease state.

### 2.6 Data management and analysis

The raw data, directly collected from the 400 donkeys, were entered into a Microsoft Excel spreadsheet and analyzed using STATA (version-13) statistical software. Descriptive Statistics were used to quantify the problems (to determine the averages and percentages) and a Chi-square (*x*^2^) test was used to determine the association of the problems/parameters with the considered risk factors (factors that predispose the donkeys to the occurrence of wounds, lameness, and other ailments. The major risk factor here is the presence and absence of intervention, i.e., the knowledge, attitude, and Practice (KAP) of the animal owners). Moreover, Mann Whitney U test was used to compare the distributions of lameness, and overall wound scores obtained from the two independent groups (i.e., donkeys in the intervention and non-intervention areas). In all calculations, the confidence level was set at 95% and statistically significant differences were considered at a value of *p* less than 0.05.

## 3 Results

### 3.1 Assessment results on the major parameters used

The prevalence of lameness and wounds on donkeys managed in non-intervention areas was higher than that in the intervention areas. Moreover, donkeys in the intervention areas had better body condition and were alert and friendly upon human approach. There was a statistically significant difference (*p* < 0.01) between the intervention and non-intervention areas in all the considered parameters, namely the presence of lameness, wounds, body condition score, demeanor, and response to approach (Table 4).

**Table 4:** Results of the assessment on the health and welfare of working donkeys in the intervention and non-intervention areas.

Variables		Intervention areas no (%)	Non-intervention areas no (%)	χ^2^	value of *p*
Wound	Present	44 (22)	75 (37.5)	11.50	0.001
	Absent	160 (79.2)	122 (61.6)		
Lameness	Present	24 (12)	51 (25.5)	11.96	0.001
	Absent	178 (88)	147 (74)		
Body condition	Poor	17 (8.5)	97 (48.5)	104.50	0.000
	Medium	93 (46.5)	85 (42.5)		
	Good	90 (45)	18 (9)		
Demeanor	Alert	165 (82.5)	136 (68)	12.11	0.002
	Dull	35 (17.5)	64 (32)		
Response to approach	Difficult to catch	43 (21.5)	67 (33.5)	7.22	0.007
	Friendly	157 (78.5)	133 (66.5)		

### 3.2 Distribution of wounds on the body of examined donkeys

The bodily distributions of the lesions are summarized in Table 5. Accordingly, the ribs, flank, hip, and back were the commonly wounded sites in donkeys living in intervention areas. On the contrary, the base of the tail, perineum, and hind legs were also more frequently wounded in donkeys from the non-intervention areas compared to the intervention areas. Except for wounds on the back and tail/tail base, there were no statistically significant differences (*p* = 0.05) in the proportion of the wounds on other body parts between the intervention and non-intervention areas.

**Table 5:** Bodily distribution of wounds on working donkeys.

Anatomical location	Intervention areas no (%)	Non-intervention areas no (%)	χ^2^	value of *p*
Back including wither	11 (5.5)	29 (14.5)	9	0.0003
Girth	3 (1.5)	6 (3)	1.02	0.31
Tail/tail base	5 (2.5)	22 (11)	11.48	0.0001
Hip and spines	13 (6.5)	11 (5.5)	0.18	0.674
Side (ribs and flank)	15 (7.5)	11 (5.5)	0.66	0.417
Perineum and hind legs	6 (3)	12 (6)	2.09	0.148

### 3.3 Severity of lameness and wounds

When comparing lameness in terms of severity in the two groups, the majority of the donkeys in the intervention areas were suffering from grade 1 and 2 lameness, but donkeys in the non-intervention areas also suffered from grade 3 and 4 lameness. Similarly, moderate and severe wounds were frequently observed on donkeys in the non-intervention areas (Table 6).

**Table 6:** Severity of lameness and wound on donkeys in the intervention and non-intervention areas.

Lameness and wound severity	Intervention areas no (%) affected	Non-intervention areas no (%) affected	Mann–Whitney *U* test	
			*Z* value	value of *p*
Severity of lameness	36,376*	43,824*	3.485	0.0005
Grade 0 (No lameness)	176 (88)	149 (74.5)		
Grade 1	9 (4.5)	17 (8.5)		
Grade 2	7 (4)	16 (8)		
Grade 3	6 (3)	9 (4.5)		
Grade 4	1 (0.5)	5 (2.5)		
Grade 5	1 (0.5)	4 (2)		
Severity of wound	37,358*	42,842*	3.999	0.0001
No wound on the body	157 (78.5)	126 (63)		
Mild would (OWS of 1 to 3)	22 (11)	26 (13)		
Moderate wound (OWS of 4 to 6)	17 (8.5)	22 (11)		
Severe wound (OWS ≥7)	4 (2)	26 (13)		

OWS, overall wound score; * Rank sum for Mann–Whitney *U* Test.

### 3.4 Presence of other signs of illness/diseases

During the physical examination of the selected donkeys, coughing, ocular discharge, colic, neurologic signs, and urogenital complications were observed with higher frequency in donkeys from non-intervention areas. However, only ocular discharge and continuous lacrimation were observed at a statistically significant level (*p* = 0.045) in the non-intervention areas compared to the intervention areas (Table 7).

**Table 7:** Common disease conditions encountered in working donkeys of the intervention and non-intervention areas.

Health problems	Intervention areas no (%) affected	Non-intervention areas no (%) affected	χ^2^	value of *p*
Coughing	13 (6.5)	17 (8.5)	0.58	0.447
Ocular discharge/continuous lacrimation	11 (5.5)	22 (11)	4	0.045
Colic	5 (2.5)	12 (6)	3.01	0.083
Neurologic signs	5 (2.5)	8 (4)	0.72	0.397
UG-complications	13 (6.5)	19 (9.5)	1.22	0.269

UG, Urogenital.

## 4 Discussion

In this study, it was revealed that the prevalence of wounds and lameness were significantly higher (*p* < 0.05) in non-intervention areas compared to intervention areas. Moreover, the majority of wounded donkeys in the non-intervention areas had more moderate to severe wounds compared to donkeys in the intervention areas. Likewise, severe lameness cases (Grade 3 and above) were also observed in donkeys in the non-intervention areas. Based on the Mann–Whitney *U* test, there was a statistically significant difference (*p* < 0.01) in the scores of lameness and wounds between the two groups, with higher scores being observed in the nonintervention areas. The difference observed in the prevalence and severity of wounds and lameness in the two areas may be due to various reasons, but the level of awareness regarding animal welfare and husbandry practice is at the forefront. Training of change agents to promote good practices (such as wound management, eye cleaning, watering, feeding, grooming, and hoof picking) and discouraging bad practices (like beating and overloading) positively changed the level of awareness and, thus, the welfare and health of the donkeys in the intervention areas. In this regard, overloading, ruthless beating, poor or improper harnessing, prolonged and improper hobbling, and overworking are usually incriminated as the causes of wounds and lameness, both in cart pulling and pack donkeys (33–37), which emanate from poor awareness of the welfare of the donkeys and the associated working animal management. Loading of hot flour from milling houses and other heavy goods on the back of pack donkeys without a saddle or with a thin saddle was also associated with back sores by the farmers in the study area. In line with this, Yilma et al. (10) and Sells et al. (30) relate pack wounds of working donkeys primarily to harnesses and load-bearing packs.

Wounds on working equines are usually developed due to poorly designed and ill-fitted harnesses, tail straps, and saddles (14). Most of the straps used in the non-intervention areas were made of strips from car tires, which cut into the skin of the equines and form large open wounds. Such wounds were seen on a greater scale in non-intervention areas compared to intervention areas, which is due to the awareness creation work implemented by non-governmental organizations. In this regard, the authors noted that fabric straps were a dominant harnessing material used in the intervention areas. Moreover, the customary use of dry sticks to beat the hindquarters and prick the tail base of a working donkey in the non-intervention areas can partly explain the greater prevalence of lesions on these parts of the body, which is in line with the findings of Pritchard et al. (15).

Donkeys in the intervention areas had moderate to good body condition scores compared to those living in non-intervention areas. Although the causes of this difference can be multi-factorial, donkeys in the intervention areas probably received better management and care, including regular deworming, sufficient feed and water, and adequate rest during and after work. Apart from its direct implication on the level of management and welfare, the body condition score can be a potential risk factor for the subsequent occurrence of wounds, which is higher in non-intervention areas compared to intervention areas. This finding is similar to Sells et al. (30), who described equines with a low body condition score as possibly having less natural padding that can protect them from pressure, friction, and shear lesions caused by harnesses. Furthermore, this is in line with Pritchard et al. (15), who identified the highest correlation coefficient (*r* = 0.37) between low body condition score and wounds in the skin and deeper tissues. From this point, it can be concluded that better working management of working equids is a basic requirement to establish or maintain good welfare standards.

Based on the behavioral assessments made, significant numbers of donkeys in the non-intervention areas were dull (32%) and difficult to catch (33.5%). This could be partly explained by the higher proportion of donkeys with poor body condition and the higher prevalence of wounds and lameness caused by overwork in these donkeys (38, 39). On the contrary, most donkeys in the intervention areas were alert (82.5%) and friendly (78.5%), indicating better behavior in their social life and interaction with humans. Unlike other behavioral tests, alertness and friendliness when approached are positive good welfare measures (15). According to Ali et al. (39), the human-equine relationship is critical to equines’ care and has implications for their psychological status. Poor welfare, which is reflected in the donkey as dullness (apathy), can be interpreted by the owner as laziness; thus, such donkeys are more likely to be beaten and suffer from chronic fear (40). Likewise, fearful and aggressive behaviors are also known to provoke a negative human reaction as owners become angry or annoyed. Therefore, animals displaying these behaviors are often exposed to adverse handling procedures because they react inappropriately to handling (41). The difference in the prevalence of wounds and lameness observed between the two study areas, as discussed above, could also emanate from the difference in the behavior of the donkeys.

Although coughing, continuous lacrimation, colic, neurologic signs, and urogenital tract complications were observed in donkeys in both areas, higher proportions of these signs were recorded in the non-intervention areas. Similarly, studies conducted earlier (24, 42–46) also indicated that respiratory problems, colic, and ocular problems were among the most prevalent health problems encountered in donkeys in Ethiopia. Moreover, Stringer et al. (14) and Berhanu et al. (27) indicated that urogenital complications are among the top 10 health problems in working donkeys in the country. Unlike lameness and wounds, significant differences were not observed in the prevalence of other health problems between the intervention and non-intervention areas.

Since work is often the only productive output of a donkey, the care given to donkeys that are unable to work properly is still unsatisfactory, both in the intervention and non-intervention areas. To bring about significant change, owners should be aware that donkeys free from injuries and any ailments can live longer and be more productive. The lower prevalence and severity of wounds and lameness, good body condition score, and positive behavior of the donkeys in the intervention areas were assumed to be due to the training provided on working animal management and the improvements to harnessing material advocated by The Donkey Sanctuary-Ethiopia. In line with this, a questionnaire survey carried out a decade ago to assess the impact of The Donkey Sanctuary on resource-poor donkey owners revealed that their donkeys were significantly healthier and more productive and, therefore, the owners were able to generate more money because of the intervention (44). A similar study conducted in West Kenya (47) using animal-based welfare assessment methods proved that non-governmental organizations had made a positive impact on donkey welfare through owner education. However, to benefit from community education or extension programs for the owners and users of equids, the knowledge transfer method should be properly designed and piloted according to the intended group (24).

## 5 Conclusion

The overall result of this study indicated that donkeys in non-intervention areas were experiencing multiple health, management, and welfare problems compared to the intervention areas. Based on the current findings, the contributions of non-governmental organizations through education programs for owners and training for vets, farriers, and allied professions are promising in the improvement of the welfare and health of working donkeys. A comprehensive and continuous equine health and welfare promotion program should be designed, introduced in the veterinary curricula, and implemented, preferably using a community-based approach to generate sustainable improvement in the welfare of working donkeys in developing countries like Ethiopia.

## Data availability statement

The raw data supporting the conclusions of this article will be made available by the authors, without undue reservation.

## Ethics statement

Before the commencement of the study, ethical approval was obtained from the Institutional Review and Ethical Board of the Faculty of Veterinary Medicine, Hawassa University (reference FVM- 287 IREB/02/2020). All the data obtained during the study were with the consent of the donkey owners. They were clearly informed about the purpose and benefit of the study.

## Author contributions

AY: Data curation, Methodology, Writing – original draft, Writing – review & editing, Investigation, Resources. DD: Data curation, Investigation, Methodology, Writing – review & editing, Formal analysis, Visualization. BM: Data curation, Methodology, Writing – review & editing.
