# Supplementary material for: Assessment of community-based intervention approaches to improve the health and welfare of working donkeys in selected areas of Sidama region, Southern Ethiopia
Source: Front Vet Sci. 2024 Jan 22;10:1253448. doi: 10.3389/fvets.2023.1253448 (PMC10839042; doi:10.3389/fvets.2023.1253448)
Supplement: Supplementary file 1 [file Data_Sheet_1.PDF]

Data collection format

1. Name of the district____________________________________
2. Name of the Kebele____________________________________
3. Are you included under NGO's (SPANA, DS, Brooke Eth.) support: Yes/No (encircle)
4. Source of the donkey: Owner/Hired/Commission (encircle)
5. How long did you spent with the donkey? (in years)_________________
6. How old are you? (in years)___________
7. Educational statues of the owner/attendant (please mark in the box):
   ☐ Illiterate ☐ Elementary school ☐ High school ☐ College(encircle)
8. Number of dependent family members: ___________________
9. Marital status (please mark in the box)
   ☐ Single ☐ Married ☐ Divorced ☐ Widowed
10. Age of the donkey (in year) __________________________
11. Sex of the donkey: Female/Male (encircle)
12. Animal work type: Cart/Pack/Both (encircle)
13. Body condition score (based on The Donkey Sanctuary's chart, 2018)
    ☐Poor (Very thin) ☐ Moderate (underweight) ☐ Ideal ☐ Overweight (fat) ☐ Obese (very fat)
14. Demure of the donkey: alert/dull (encircle)
15. Response of the donkey to approach by unfamiliar person:

☐	difficult to catch
☐	friendly

16. Response of the donkey when someone walk around:
    ☐ No response ☐ tucks tail
    ☐ moves away ☐ aggression
17. Wound on the body of the donkey: Present/Absent (encircle)
18. If wound present,
    a) Severity and number (count all the wounds and put them by severity)

Wound score (based on Sells et al, 2010)	Superficial (1)	Medium(2)	Deep (3)
Number of wound			

    b) Bodily distribution of the wound (Where on the donkey?)________________________________
    c) What was the major source/cause of the wound?______________________________________
19. Is the donkey lame? Yes/no (encircle)
    a) If yes, please mare on the observed sign/indicators (please mark in the box)

☐ Sickle hock conformation	☐ abnormal foot placement
☐ Pain on palpation	☐ pain on percussion of the hoof walls
☐ Swelling of specific limb structures	☐ Superficial Lesion on the articular area
☐ Pain and stiffness on flexion of limb joints	☐ skeletal asymmetry of the hindlimbs and hindquarters
☐ resistance to lumbar spinal flexion	☐ pain on flexion of the cervical and thoracolumber spine

    b) If yes, what was the severity of the lameness? (Scored from Grade 0 to Grade 5 based on **AAEP 1991**) (please mark in the box)
    ☐ Grade ☐ Grade
    ☐ Grade ☐ Grade

☐ Grade ☐ Grade

20. Was the donkey sick in the past one month? Yes/No
    i. If yes, What were the major signs/problem? (please mark in the box)
       - ☐ Coughing and/or nasal discharge
       - ☐ Continuous lacrimation/ocular discharge
       - ☐ Abdominal pain, rolling and frequent stretching (suggestive of colic)
       - ☐ African horse sickness
       - ☐ Tetanus
       - ☐ Strangles
       - ☐ Nervous signs (such as head pressing, imbalance, depression, excitation etc)
       - ☐ Urogenital problems (Difficulty of urination, frequent urination, pain during urination, blood in the urine, etc)
       - ☐ Dermatological problems (including sarcoids)
       - ☐ Epizootic lymphanhgitis
       - ☐ Others, specify_____________________________________
    ii. If yes, what have you done to help your sick donkey? (please mark in the box)
       - ☐ Took to the veterinary clinic
       - ☐ Purchased medicine from the local veterinary drug vendor and administered by self
       - ☐ Visited the nearby NGO working on equine health and welfare
       - ☐ Consulted the nearby traditional healer
       - ☐ Did nothing
       - ☐ Other, specify ____

:
